# WLS-ILIAD: Initial Lifetime’s Impact on ADRD

**DOI:** 10.1093/geroni/igab046.851

**Published:** 2021-12-17

**Authors:** Pamela Herd

**Affiliations:** Georgetown University, Georgetown University, District of Columbia, United States

## Abstract

Between 2021 and 2025, WLS will collect two new waves of data, which will capture detailed measures of cognitive change and dementia as the cohort reaches their early to mid 80s. In this session, I will provide an overview of the data that we’re collecting, as well as opportunities to explore early and mid-life determinants of cognitive change and dementia onset in this unique study. Compared to existing studies, the WLS offers some novel opportunities. First, it will provide one of the only opportunities to study how early and midlife life conditions and experiences, on data gathered prospectively, can shape cognitive trajectories and dementia in later life. Second, its unique sibling design provides significant analytic advantages, improving causal inference. Third, the study includes a large group of rural participants, allowing for closer examinations of how rural conditions may shape risk and resilience against cognitive decline and dementia in later life.

